# Metformin sensitizes chemotherapy by targeting cancer stem cells and the mTOR pathway in esophageal cancer

**DOI:** 10.3892/ijo.2014.2450

**Published:** 2014-05-21

**Authors:** SOICHIRO HONJO, JAFFER A. AJANI, AILING W. SCOTT, QIONGRONG CHEN, HEATH D. SKINNER, JOHN STROEHLEIN, RANDY L. JOHNSON, SHUMEI SONG

**Affiliations:** 1 Department of Gastrointestinal Medical Oncology, University of Texas M.D. Anderson Cancer Center, Houston, TX, USA; 2 Department of Radiation Oncology, University of Texas M.D. Anderson Cancer Center, Houston, TX, USA; 3 Department of Gastroenterology, Hepatology and Nutrition, University of Texas M.D. Anderson Cancer Center, Houston, TX, USA; 4 Department of Biochemistry and Molecular Biology, University of Texas M.D. Anderson Cancer Center, Houston, TX, USA

**Keywords:** metformin, cancer stem cells, mTOR, chemotherapy

## Abstract

Our clinical study indicates esophageal adenocarcinoma patients on metformin had a better treatment response than those without metformin. However, the effects of metformin and the mechanisms of its action in esophageal cancer (EC) are unclear. EC cell lines were used to assess the effects of metformin alone or in combination with 5-fluorouracil on survival and apoptosis. RPPA proteomic array and immunoblots were used to identify signaling affected by metformin. Standard descriptive statistical methods were used. Reduction in cell survival and induction of apoptosis by metformin were observed in several EC cell lines. The use of metformin in combination with 5-FU significantly sensitized EC cells to the cytotoxic effect of 5-FU. RPPA array demonstrated that metformin decreased various oncogenes including PI3K/mTORsignaling and survival/cancer stem cell-related genes in cells treated with metformin compared with its control. Immunoblots and transcriptional analyses further confirm that metformin downregulated these CSC-related genes and the components of the mTOR pathway in a dose-dependent manner. Sorted ALDH-1+ cell tumor sphere forming capacity was preferentially reduced by metformin. Finally, metformin reduced tumor growth *in vivo* and when combined with FU, there was synergistic reduction in tumor growth. Metformin inhibits EC cell growth and sensitizes EC cells to 5-FU cytotoxic effects by targeting CSCs and the components of mTOR. The present study supports our previous clinical observations that the use of metformin is beneficial to EC patients. Metformin can complement other therapeutic combinations to effectively treat EC patients.

## Introduction

Esophageal cancer (EC) is a lethal illness with high incidence globally and significant numbers of new cases in the USA (1–3). If it is diagnosed as localized cancer, it is often treated with chemoradiation therapy plus minus surgery (depending on the extent of the cancer and whether patient can withstand surgery) (4,5), however, finding residual cancer in the surgical specimen (6,7) or high rate of relapse or persistence of cancer is common (8). This suggests that EC is an inherently resistant cancer. We have previously reported that hedgehog (Hh) pathway is often upregulated in EC and mediates therapy resistance (9,10). We also reported that mTOR pathways is often activated and can be the source of secondary resistance to Hh inhibition (11,12). Intriguingly, the mTOR pathways along with Hh (and other) pathways have been implicated in the maintenance of cancer stem cells (CSCs) (13–17).

There has been considerable interest in the anti-diabetic agent metformin that induces AMPK-dependent inhibition of IGF-1 ultimately leading to inhibition of mTORC1. Metformin also silences mTORC1 through LKB1 and eventually it is able to shut down ERK1/2 (18). Loss of LKB1 is known to confer aggressive phenotype to some esophageal cancer cells (19). We previously reported a retrospective analysis of esophageal adenocarcinoma (EAC) patients who were on metformin for diabetes and compared their outcome with those EAC patients who were not taking metformin (20). In this small cohort comparison, patients who were taking metformin had better response to chemoradiation therapy.

Taking the literature and our clinical experience with metformin in EAC together, we have carried out a number of non-clinical experiments to document metformin’s role in EC CSCs and the mTOR pathway and demonstrated that metformin can complement other traditional therapies to effectively treat EC patients.

## Materials and methods

### Cells and reagents

The human EAC cell lines FLO-1, BE3, SKGT-4, OE33, JHESO and OACP were kindly provided by Dr Uma Raja and Dr Mien-Chie Hung [University of Texas (UT) M.D. Anderson Cancer Center, Houston, TX, USA] and have been previously described (21,22). The human esophageal squamous carcinoma (SCC) cell lines-YES-6 and KATO-TN were kindly provided by Dr Health Skinner (UT M.D. Anderson Cancer Center). These cell lines were authorized and re-characterized in the characterized cell line core facility of UT M.D. Anderson Cancer Center every 6 months. Metformin was obtained from Calbiochem (San Diego, CA, USA). 5-FU was obtained from Sigma (St. Louis, MO, USA). Antibodies phospho-AKT, phospho-S6 (235), phospho-70S6, Jagged1 and MCL were purchased from Cell Signaling (Beverly, MA, USA). The antibodies Shh and ALDH1 were from Abcam (Cambridge, MA, USA). YAP1 was purchased from Santa Cruz Biotechnology (Santa Cruz, CA, USA); SOX9 was purchased from Chemicon (Billerica, MA, USA).

### Cell proliferation assay

Cell proliferation assays were performed using the Cell Titer 96 aqueous non-radioactive cell proliferation assay (MTS) according to the instructions of the manufacturer (Promega Co., Madison, WI, USA). All assays were performed in triplicate and repeated at least three times.

### Flow cytometric labeling and fluorescence-activated cell sorting

ALDH1 activity was assessed by fluorescence-activated cell sorting in EAC cell line JHESO according to the ALDEFLUOR based cell detection kit (Stemcell Technologies Inc, Vancouver, BC, Canada) following the protocol and Diethylaminobenzaldehyde (DEAB) was used to inhibit ALDH-1 activity to show the specificity of the detection. ALDH1 positive or negative cells were sorted from JHESO EAC cells by fluorescence-activated cell sorting according to the ALDEFLUOR detection kit. ALDEFLUOR/DEAB treated cells were used to define negative gates. FACS was performed with >1×10^6^ cells using the BD FACSCanto II (Becton-Dickinson, Franklin Lakes, NJ, USA) or FACSAria (Becton-Dickinson).

### Tumorsphere formation assay

Sphere culture was performed as previously described (24). Briefly, Single EAC cells or FACS-isolated ALDH1+ or ALDH1− cell populations (1,000/well) were seeded in triplicate onto a 6-well ultra-low attachment plate. After 10–14 days of culture, the number of tumorspheres formed (diameter >100 μm) was counted under microscope.

### Protein extraction and western blot analysis

Protein isolation and western blot analyses were performed as previously described (25).

### Reverse-phase protein arrays (RPPA)

RPPA analysis was performed in cell lysate from JHESO cells control and treated with 10 mM metformin for 48 h in RPPA core facility, the UT M.D. Anderson Cancer Center. Samples were serially diluted 2-fold for 5 dilutions and probed with 175 antibodies and arrayed on nitrocellulose-coated slides. Relative protein levels were normalized for protein loading and determined by interpolation of each dilution curve from the standard curve as previously described (28).

### Transient transfection and luciferase reporter assays

Jagged1 luciferase promoter construct was provided by Dr Randy Johnson and has been previously described (23). SOX9 luciferase promoter construct has been previously described (24). Transient co-transfection with luciferase reporters and *Renilla* vector were performed as previously described (24).

### Indirect immunofluorescence and flow cytometry

Indirect immunofluorescence staining was performed as described (25). Putative cancer stem cells was labeled by indirect anti-OCT4 antibody and anti-ALDH1 at 1:100 and analyzed by flow cytometry using BD FACSCalibur (BD Biosciences, Franklin Lakes, NJ, USA).

### Flow cytometric and apoptotic analysis

Flow cytometric analysis was performed as described (24). In briefly, SKGT-4 and Yes-6 cells were seeded onto 6-well plates (1×10^5^ per well) in DMEM and cultured for 24 h to allow cell attachment. The cells were then treated with 0.1% DMSO or metformin at 10 mM, 5-FU at 10 μM or in combination of both for 48 h. The cells were then harvested, fixed with methanol, washed, treated with RNase A, and stained for DNA with propidium iodide (Sigma) and then were analyzed for DNA histograms and cell cycle phase distribution by flow cytometry using a FACSCalibur instrument (Becton-Dickinson). To determine whether the cells treated with metformin underwent apoptosis, cells treated with up to 10 mM metformin for 48 h and washed in PBS, resuspended in 100 μl of binding buffer containing FITC-conjugated Annexin V, and analyzed by flow cytometry to determine the apoptosis index.

### Xenograft mouse model

JHESO EAC cells were subcutaneously injected with 2×10^6^ cells in nude mice. When tumors reached a size of approximately 50 mm^2^, mice were divided by four groups: buffer alone (control), metformin (200 μg/ml) in drinking water daily, 5-FU at 20 mg/kg/mouse was treated by i.p. injections and the combination of metformin and 5-FU. n=5 for each group. The tumor size was measured by using a digital caliper (VWR International, Radnor, PA, USA), and the tumor volume was determined with the formula: tumor volume [mm^3^] = (length [mm])^*^(width [mm])^2*^0.52. All the measurements were compared using unpaired Student’s t-test.

### Statistical analysis

Data were analyzed using the Student’s t-test. A P-value of <0.05 was required for statistical significance, and all tests were two-sided. All tests were done with SPSS 10.1 software (SPSS, Inc., Chicago, IL, USA).

## Results

### Metformin inhibits tumor cell growth and sensitizes chemotherapy in human esophageal cancer cells

**Figure 1:**
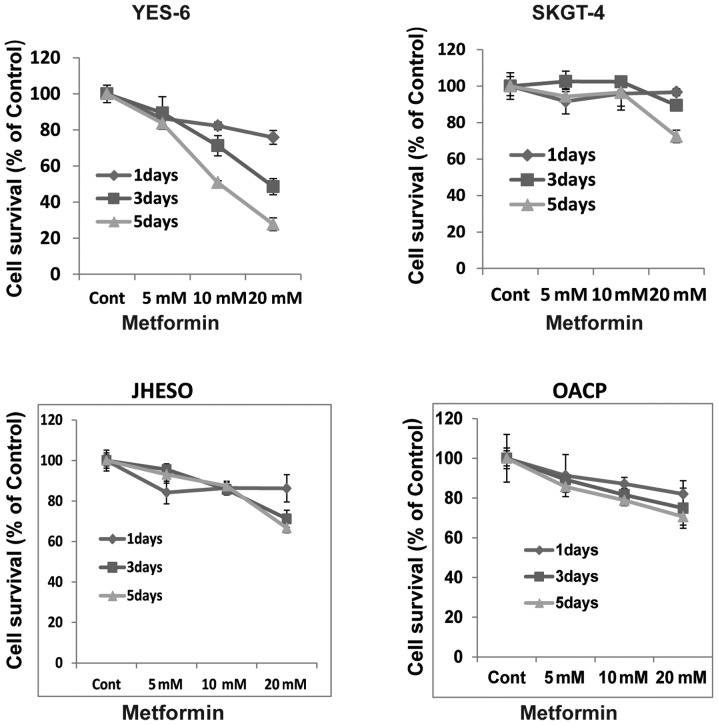
Metformin inhibits EC cell growth. Esophageal cancer cells SKGT-4, JHESO, OACP and YES-6 were seeded in 96-well plates and treated with up to 20 mM metformin for 1, 3 or 5 days. A non-radioactive MTS cell proliferation assay was done to determine the rate of proliferation as described in Materials and methods. The experiments were performed in triplicate and repeated twice; the results reported here are the mean of SD of all these experiments.

**Figure 2:**
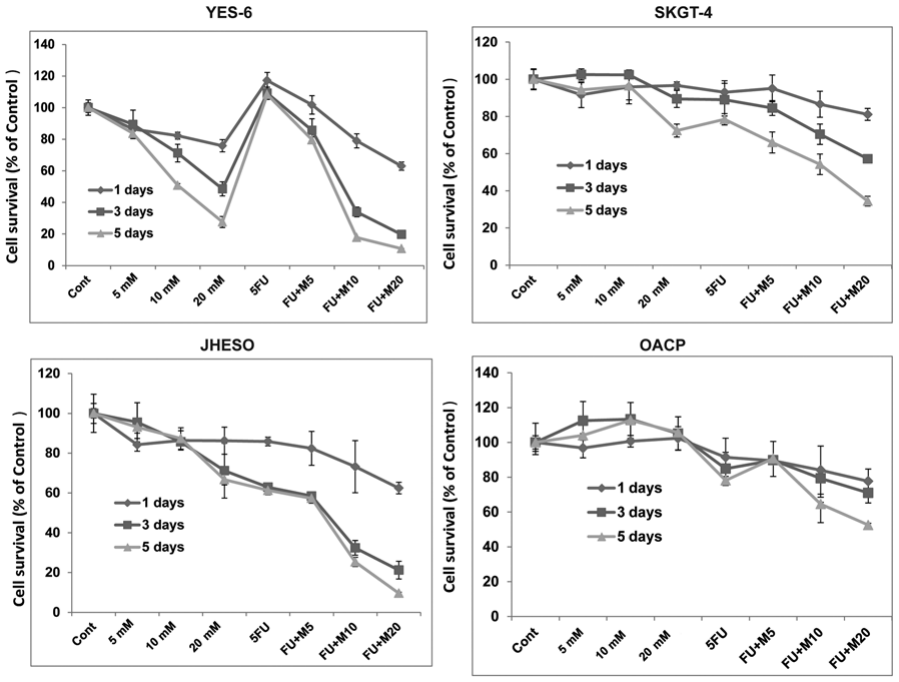
Metformin enhances 5-FU sensitivity on EC cells. Esophageal cancer cells SKGT-4, JHESO, OACP and YES-6 were seeded in 96-well plates and treated with metformin alone at indicated dosage, 5-FU alone at 10 μM and the combination of both for different time points. A non-radioactive MTS cell proliferation assay was done to determine the rate of proliferation (cell survival). The experiments were performed in triplicate and repeated twice; the results reported here are the mean of all these experiments.

To evaluate the effects of the growth activity of metformin on human esophageal cancer cells *in vitro*, we examined the effects of metformin on four EC cell lines, YES-6, SKGT-4, JHESO and OACP. A dose- and time-dependent inhibition of cell proliferation was observed in YES-6, OACP cells and JHESO cells but less effective in SKGT-4 (Fig. 1). However, synergistic inhibition of cell proliferation was noted in all these cell lines when metformin in combination with 5-FU indicating metformin sensitize 5-FU on inhibition of EC cell growth (Fig. 2).

### Metformin induces apoptosis in EC cells

**Figure 3:**
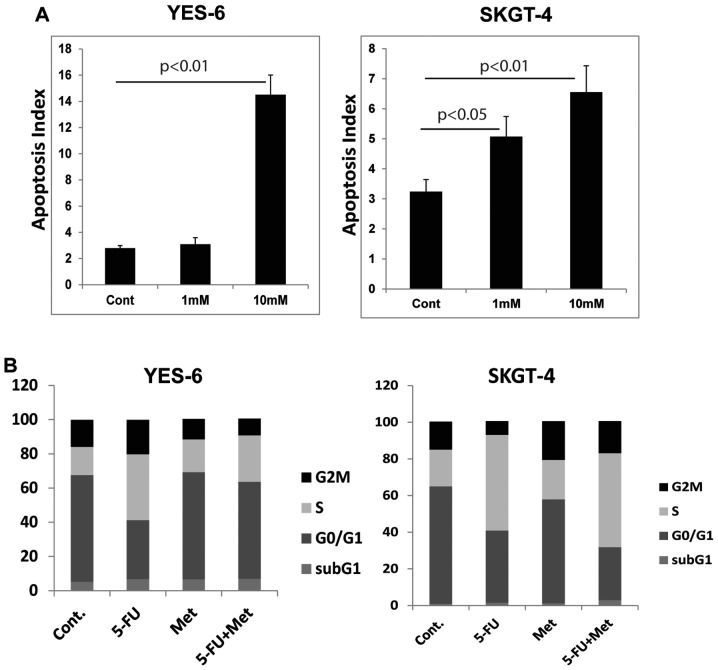
Metformin induces apoptosis and changes in the cell cycle. (A) Yes-6 and SKGT-4 cells were seeded in 6-well plates and treated with 0.1% DMSO or with 1 or 10 mM metformin for 24 h and resuspended in 100 μl of binding buffer containing FITC-conjugated Annexin V, and analyzed by flow cytometry to determine the apoptosis index. (B) The cells were fixed and stained for DNA with propidium iodide and then analyzed for DNA histograms and cell cycle phase distribution by flow cytometry using a FACSCalibur instrument.

Metformin increase the number of cells with apoptosis in a dose-dependent manner in YES-6 cells and SKGT-4 cells (Fig. 3A). 5-FU alone dramatically increased S phase arrest in these EC cells (Fig. 3B), while the combination of metformin and 5-FU reduced the proportion of S phase cells and increase the apoptotic cell population.

### Metformin suppresses CSC-related gene expression in EC cells

**Figure 4:**
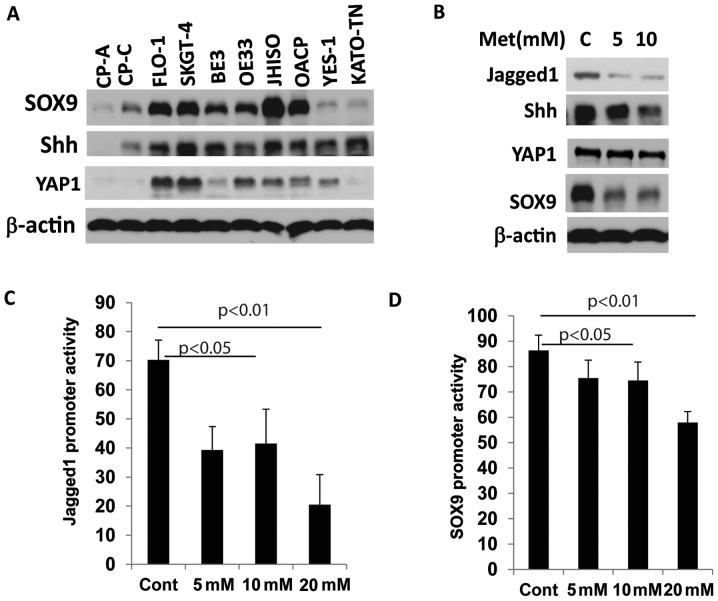
Metformin inhibits stem cell signaling related genes in EC cells. (A) Immunoblots were done with antibodies against SOX9, Shh and YAP1 in eight esophageal cancer cell lines and two immortalized Barrett’s cell lines (CP-A and CP-C). (B) SKGT-4 cells were treated with 5 or 10 mM metformin for 24 h and immunoblots were done with the antibodies indicated in part B. (C and D) Yes-6 and JHESO cells were cotransfected with Jagged1 and SOX9 luciferase reporter and pCH110 and then challenged with metformin at indicated dosage. Luciferase reporter activity was measured after 24 h. Columns, mean of at least three independent experiments; bars, SD.

Metformin as a CSC targeting agent in breast cancer and ovarian cancer has been previously reported (17,26). We sought to determine whether metformin acts on CSCs population and CSC-related genes in EC cell lines. We therefore first assessed whether CSC-related genes are elevated in EC cell lines. Eight cancer cell lines and two Barrett’s esophagus (BE) cell lines were immunoblotted for the expression of CSC- related genes SOX9, YAP1 and Shh (Fig. 4A). SOX9, Shh and YAP1 were overexpressed in many cancer cell lines compared to BE cell lines (CP-A and CP-C) indicating the activation of stem cell signaling in EC cells. When metformin was exposed at increasing concentrations, expression of the CSC-related genes SOX9, Jagged1 and Shh were greatly reduced (Fig. 4B).

We then explored whether metformin affects transcription of CSC-related genes by affecting their promoter activity. In YES-6 and JHESO cells, co-transfected with Jagged1 or SOX9 luciferase reporter and pCH110 as an internal control vector were exposed to various concentration of metformin. Luciferase reporter activity was measured after 24 h and demonstrated a dose-dependent reduction in both of Jagged1 and SOX9 promoter activities in EC cells in concert with their reduction in expression levels (Fig. 4C and D).

### Metformin targets EC cancer stem cells (CSCs)

**Figure 5:**
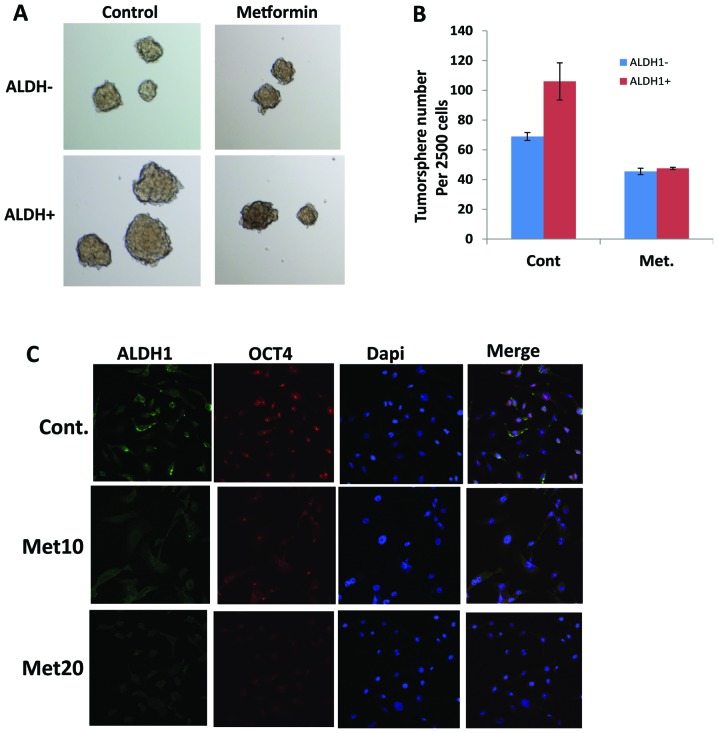
Metformin preferentially inhibits tumor sphere formation in ALDH1+ EC cells. (A and B) ALDH1 positive or negative cells were sorted from JHESO EA cells by fluorescence-activated cell sorting according to the ALDEFLUOR detection kit. Tumor sphere assays were done in the sorted cells in triplicate in ultra-low attachment plate in tumor sphere medium. After 8–10 days of culture, the tumor sphere numbers were counted under a microscope. Representative fields and the bar graph are shown in (A and B). (C) ALDH1 and OCT4 expression and localization were analyzed by immunofluorescence as described in Meterials and methods.

Given metformin as an effect agent targeting CSC-related genes, we investigated the impact of metformin on EC CSCs. We have experienced that ALDH1 is a reliable CSC marker in EC tumor cells and CSCs are characterized by their ability to form tumor spheres in suspension in serum-free medium. Therefore, JHESO cells were first sorted to separate ALDH1+ and ALDH1− cells. Tumor sphere forming capability was studied in the JHESO ALDH1+ and ALDH1− cells. Results in Fig. 5A demonstrate that ALDH1+ cells formed larger tumorspheres but this capability was significantly reduced by metformin (Fig. 5A and B). Metformin also reduced the fraction of ALDH1+ cells in the JHESO cells (data not shown). In addition, metformin suppressed the CSC markers ALDH-1 and OCT4 in a dose-dependent manner by immunofluorescence (Fig. 5C). These data indicate that metformin targets EC CSCs.

### RPPA proteomic analysis on metformin treated JHESO cells and in combination of 5-FU and metformin on CSC-related genes and mTOR components

**Figure 6:**
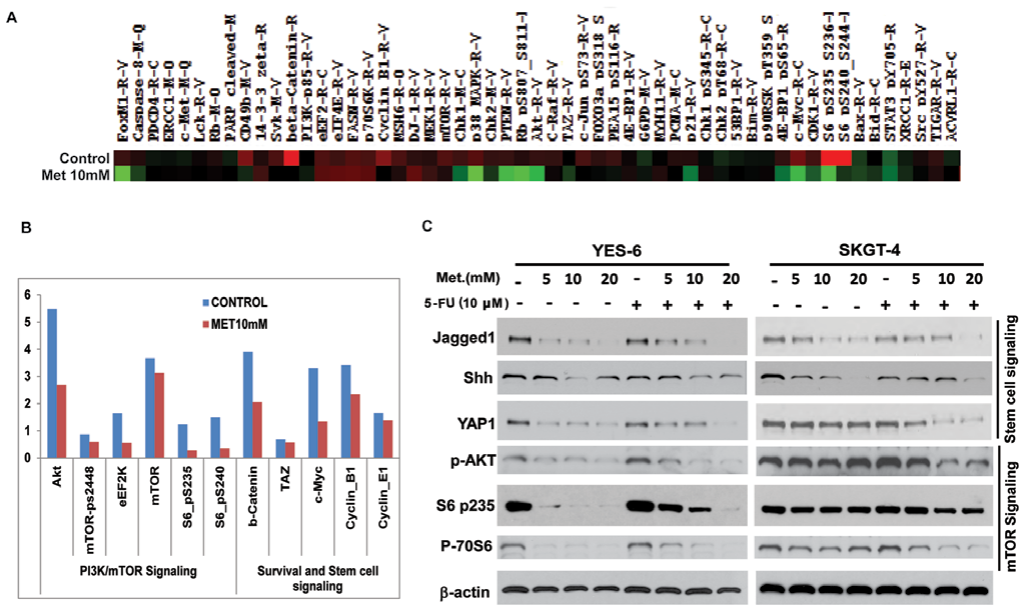
RPPA proteomic analysis on metformin treated JHESO cells and the effects of metformin on mTOR pathway and stem cell signaling. (A) Heat map representation of RPPA analysis showing gene expression changes in parental and metformin (10 mM for 48 h)-treated JHESO cells. (B) Expression level change in selected genes after normalization by RPPA analysis. (C) SKGT-4 and YES-6 cells were treated with metformin alone at indicated dosage, 5-FU alone at 10 μM and in combination of both for 24 h and immunoblots were done with the antibodies involving in stem cell signaling (Jagged1, Shh and YAP1) and mTOR pathways (p-AKT, p-S6, p70S6) as indicated in the figure.

We next sought to define the mechanisms by which metformin decreased cell growth and sensitize EC cells to 5-FU. Cell lysate from JHESO cells treated with metformin at 10 mM for 48 h and its control was performed RPPA proteomic array assays. As demonstrated in Fig. 6A, many oncogenic genes were downregulated by treatment of metformin. As shown in Fig. 6B, the most reduced ones are proteins in the PI3K/mTOR signaling including phospho-S6-p-235 and phospho-S6p-240 and AKT and genes (β-catenin and C-MYC) in stem cell signaling.

To further confirm whether these genes were downregulated by metformin and in combination of chemo-agent 5-FU in EC cell lines, western blot analyses were performed in YES-6 and SKGT-4 cells. As shown in Fig. 6C, metformin alone strongly decreased the expression of stem cell signaling markers (Jagged1, Shh, YAP1) and mTOR pathway components-phospho-AKT, phosphor-S6, phosphor-70S6 in a dose-dependent manner. The addition of 5-FU to different dosage of metformin further reduced expression of various CSC related genes (Jagged1, Shh, YAP1) and mTOR (p-AKT, p-70S6 and pS6) components in both EC cell lines indicating metformin inhibits EC cell proliferation and sensitize cells to 5-FU by targeting both CSCs and mTOR pathways.

### Metformin inhibits tumor growth especially when in combination with 5-FU in vivo

**Figure 7:**
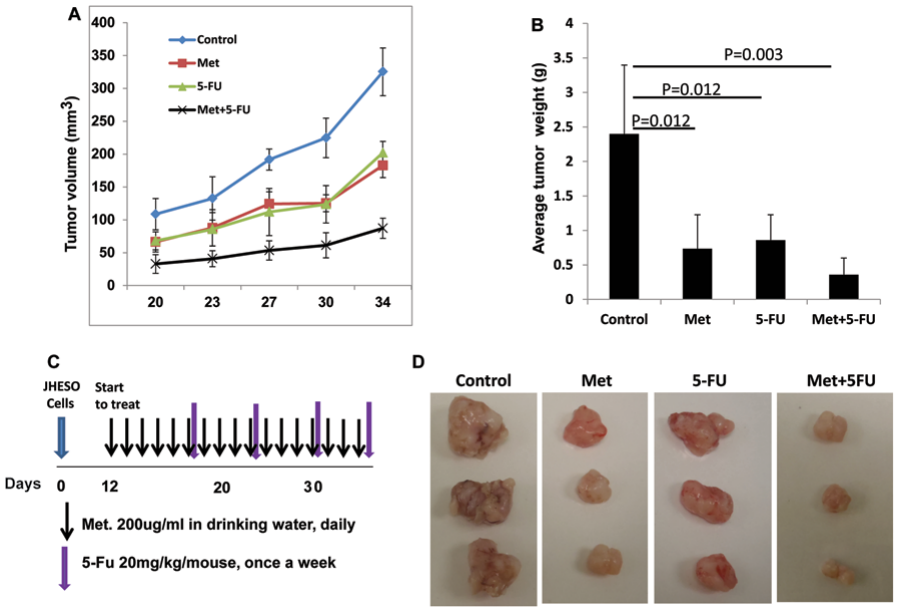
Metformin synergizes 5-FU in reducing EC tumor growth *in vivo*. JHESO cells were inoculated into nude mice (n=5 per group) at 2×10^6^ per mouse subcutaneously. (A) Tumor volume and (B) weight were calculated as indicated in Materials and methods. (C) Diagram demonstrate the treatment route and dosage and frequency for metformin and 5-FU. (D) Representative tumors in each group are demonstrated.

JHESO cells were inoculated into the nude mice at 2×10^6^ cells per mouse subcutaneously and treated with metformin or 5-FU alone and metformin plus 5-FU (schema of therapy shown in Fig. 7C). Results from *in vivo* xenograft model further confirmed that metformin or 5-FU alone reduced tumor volume and weight (Fig. 7A and B). The combination of metformin and FU resulted in synergistic reduction in the tumor volume and tumor weight (Fig. 7).

## Discussion

It has been implicated that metformin, commonly used as an oral anti-hyperglycemic agent, may reduce cancer risk and have antitumor effects in many types of cancer. Our previous study demonstrated that metformin treatment improved the response to neoadjuvant chemoradiation in esophageal adenocarcinoma patients (1). However, the effects of metformin and in combination of chemotoxic agents on both ESCC and EAC and their mechanisms of action remain unclear. In this study, we demonstrated that metformin inhibit cell growth in both ESCC and EAC cells and sensitize 5-FU cytotoxic effects by targeting CSCs and mTOR signal pathways.

Our results are consistent with the study of Kobayashi *et al* (32) regarding the antitumor effects of metformin on ESCC cell lines *in vitro*. However, our study demonstrated that metformin acts effectively not only on ESCC cells but is also effective on EAC cells by using both ESCC and EAC cell lines, indicating a therapeutic view, metformin has equal effects on both ESCC and EAC. Most importantly metformin sensitizes the cytotoxic agent (5-FU) on both types of EC cells and inhibits the growth of EC cells *in vitro* and in a xenograft nude mouse model. This suggests that metformin can act as an important auxiliary drug to improve the EC patients’ survival.

EC is a difficult cancer to treat because it is often resistant to the current standard therapies. The reason for this inherent resistance is likely the genetic make-up of EC. ECs have one of the highest genetic alterations (insertion, deletion, mutation, amplification and or recombination) rates and each EC can have as many as 50 or higher non-synonymous alterations (27). It is also suggested that CSCs may play a central role in imparting resistance to therapy and that the density of CSCs has a role as well (28). Our previous data are supportive of the concept that CSCs are animated upon chemotherapy or radiation injury to EAC in a xenograft model and the ‘first responders’ are cells with CSC markers such as Gli-1 and Shh (9). Our current data suggest that the proportion of ALDH-1+ cell fraction varies among the EAC cell lines and cells with higher proportion of ALDH-1+ cells have more potential to form tumor spheres and tend to be resistant to therapy. Much of our findings are consistent with those demonstrated in other tumor types, however (13,14,16,17), the novelty of our findings include demonstration that CSC-associated group of genes are upregulated in EC tumor cells and metformin suppresses these genes and effectively decrease ALDH1+ CSCs tumor sphere formation, indicating a metformin targeting CSC population in EC cells.

Amplification or mutations in the RTK-PI3K-mTOR pathway have been identified by whole genomic sequencing, whole exome sequencing and high-density genomic profiling arrays (29,30) in EC tumors. Mutations were discovered in 23% of EC tumor, with PIK3CA/mTOR being the most frequently mutated (30). In addition, there is upregulation of mTOR components in EC tumors especially the resistant population and overexpression of mTOR associated with poor survival in EAC (31). Therefore, mTOR might be an important therapeutic target and should be considered a priority in the therapeutic strategies in EC patients. Our study demonstrated that metformin effectively downregulates mTOR components including phospho-AKT, phospho-S6, phospho-70S6 which are important factors maintain tumor cell growth. Taken together, metformin suppresses EC cell growth *in vitro* and *in vivo* due to its ability to reduce the CSCs population as well as causing inhibition of the mTOR pathway in bulk tumor cells. Further, the synergy between metformin and 5-FU is particularly of interest because it would potentially afford an opportunity to treat the CSCs and proliferating component simultaneously to increase the sensitivity of chemoradiation in EAC patients.

In conclusion, our non-clinical results are supportive of our prior retrospective observations in the clinic that patients who were taking metformin (for diabetes) had better therapy outcome than those who were not taking metformin, and we find metformin inhibited the EAC cell growth and increased the sensitivity to 5-FU cytotoxic effects by targeting the genes of CSCs and mTOR signal pathways. Considerably more work is necessary to control the CSC population in EAC that can prevent repopulation of the tumor bed after therapy.
